# Rutin Maintains the Thermogenic Phenotype of Beige Adipocytes and Concomitantly Suppresses Mitophagy Against Obesity in HFD Mice

**DOI:** 10.3390/metabo16010012

**Published:** 2025-12-23

**Authors:** Jianmei Li, Kexin Li, Shengnan Li, Jingxun Cui, Shuangying Zhou, Huiwen Wu

**Affiliations:** 1 Graduate School, Shanxi Medical University, Taiyuan 030001, China; yililimeizi@163.com (J.L.); 14794827543@163.com (K.L.); lisnnnnn@163.com (S.L.); 2 Science and Technology Center, Shanxi University of Medicine, Fenyang 032200, China; 3 Department of Clinical Medicine, Shanxi University of Medicine, Fenyang 032200, China; cuajixny@163.com (J.C.); zhousy00_00@163.com (S.Z.)

**Keywords:** obesity, beige adipocytes, rutin, mitophagy

## Abstract

Background: The browning of white adipose tissue for thermogenesis is an effective strategy for combating obesity. The formation of beige adipocytes is reversible, making their maintenance a key therapeutic target. Rutin has been shown to promote the transition from white to beige adipocytes. It remains unclear whether rutin can prevent the reversion of beige adipocytes to white adipocytes and what mechanisms underlie this process. Objectives: This study aims to determine whether rutin can sustain the thermogenic phenotype of beige adipocytes and to elucidate its mechanism. Methods: We established a beige adipocyte model with CL-316, 243(CL) in vitro. A white adipocyte model was created by CL withdrawal after 3 days. Then, we conducted a co-intervention with CL and rutin, as well as sustained rutin intervention on beige adipocytes following CL withdrawal. In vivo, we utilized a C57BL/6 mouse model, including ND, high-fat diet (HFD), and HFD + Rutin groups. The mice were further divided into Cold and −Cold groups, with the former undergoing 7 days of exposure to 4 °C and the latter experiencing 10 days of 22–24 °C. Rutin was administered continuously until the conclusion of the experiment. Results: Rutin consistently ameliorates metabolic disorders and prevents the expansion of adipose tissue. It concomitantly suppresses mitochondrial autophagy during beige induction, upregulates thermogenic markers in brown adipocytes, and safeguards the mitochondrial-related functional indicators. Conclusions: In summary, rutin obstructs the transformation of beige adipocytes into white adipocytes and concomitantly suppresses mitochondrial autophagy, thereby continuously improving obesity induced by a high-fat diet.

## 1. Introduction

In recent years, the number of patients with obesity has continued to rise worldwide, becoming a global epidemic [1]. Research indicates that obesity contributes to increased prevalence of type 2 diabetes, cardiovascular disease, and non-alcoholic fatty liver disease [2]. The primary driver of obesity is an imbalance between caloric intake and energy expenditure, which facilitates the expansion of adipose tissue [3]. As the body’s largest energy reservoir, adipose tissue plays a crucial role in regulating systemic energy balance and metabolism [4]. Adipose tissue comprises white adipose tissue (WAT), brown adipose tissue (BAT), and beige adipocytes [5]. BAT is responsible for active non-shivering thermogenesis during infancy and early childhood. However, both the function and mass of BAT decline with age, and its activity is further diminished in conditions such as obesity, diabetes, and aging [6,7]. Certain white adipocytes residing within WAT depots respond to cold exposure or other stimuli by forming multi-vesicular lipid droplets and accumulating a number of mitochondria [8]. Beige adipocytes exhibit thermogenic functions similar to those of brown adipocytes when they are activated. Consequently, they represent a promising target for obesity treatment.

The most notable shared characteristic of beige and brown adipocytes is their high mitochondrial content and the presence of uncoupling protein 1 (UCP1). UCP1 is found in the inner mitochondrial membrane that participates in proton leakage. This protein disrupts the electrochemical gradient across the inner mitochondrial membrane, thereby decoupling mitochondrial ATP synthesis. ATP production is inhibited, leading to energy dissipation as heat and increased metabolic rate [7,9]. β3 receptors are activated with the β3-adrenergic receptor agonist or rosiglitazone following chronic cold exposure. They then bind to peroxisome proliferator-activated receptor gamma (PPARγ), which is a key factor in adipocyte differentiation. This interaction shifts PPARγ regulation from promoting “white adipocyte differentiation” to facilitating “beige adipocyte differentiation” [10]. The upregulation of PR domain-containing 16 (PRDM16) and peroxisome proliferator-activated receptor γ coactivator 1alpha (PGC-1α) enhances UCP1 expression and mitochondrial biogenesis, collectively driving the transcriptional program of brown adipogenesis [11]. When these stimuli diminish, not only do UCP1 and mitochondrial function gradually decline, but beige adipocytes revert to white adipocytes. This phenomenon is referred to as the beige-to-white transition [12,13]. Therefore, promoting the expression of beige adipocytes and sustaining their functionality presents significant therapeutic potential for combating excess fat accumulation in populations affected by obesity and diabetes induced by HFD.

Mitochondria are crucial cellular organelles responsible for energy production. Energy imbalance in obesity is associated with adipocyte dysfunction caused by mitochondrial homeostasis disruption [14]. Beige adipocytes regulate mitochondrial content through mitochondrial biogenesis and mitochondrial autophagy, which maintain cellular mitochondrial homeostasis [15]. Previous studies have demonstrated that brown adipocytes are formed in response to external stimuli like cold exposure [16]. They then directly revert to white adipocytes through mitochondrial degradation mediated by mitochondrial autophagy once the stimulus is withdrawn and the environmental temperature returns to room temperature. In a study conducted by Lu X and Altshuler-Keylin S et al., *Park2*-knockout mice demonstrated a significant reduction in inguinal WAT (iWAT) mass compared to control mice 15 days after the withdrawal of the β3-adrenergic receptor agonist CL [17]. Nevertheless, iWAT in the *Park2*-knockout mice retained a substantial population of UCP1^+^ beige fat cells, while the control mice exhibited a loss of recruited beige adipocytes [17]. In certain studies, pharmacological interventions inhibited PINK1-Parkin-mediated mitochondrial autophagy, thereby preserving beige adipocyte thermogenesis. Following damage or decline in mitochondrial membrane potential, PINK1 accumulates on the outer mitochondrial membrane and subsequently recruits Parkin, a multifunctional ubiquitin ligase, to ubiquitinate PINK1 [18,19]. Subsequently, selective autophagy adaptor proteins such as NDP52, NBR1, OPTN, and p62 (SQSTM1) link ubiquitinated mitochondrial proteins to microtubule-associated protein light chain 3(LC3), forming autophagosomes that target damaged mitochondria for degradation in lysosomes [20,21]. By downregulating PINK1 and upregulating p62 expression through the AMPK-Drp1 pathway, Wang et al. suggested that quercetin 3-O-glucuronide (Q3G), a key flavonoid in lotus leaf extract (FNE), stimulates beige adipocyte gene expression and causes beige induction [22]. In other people’s research, intervention with Liensinine (Lie) in adipocytes after rosiglitazone (Rosi) withdrawal revealed that Lie blocked mitochondrial autophagy flux, thereby preserving beige adipocyte characteristics [23]. Accordingly, medications that block mitochondrial autophagy can maintain the thermogenic effects of browning and maintain the traits of beige adipocytes after external stimuli subside [23,24,25,26].

Rutin, a natural flavonoid substance widely present in various plants, possesses multiple functions, including anti-inflammatory and anti-obesity effects. It is considered one of the top therapeutic active compounds [27]. Early studies demonstrated that rutin improves obesity and hyperlipidemia induced by HFD in rats and mice [28]. Previous research from our group also confirmed rutin’s ability to improve obesity in HFD mice and showed that it can upregulate the expression of Ucp-1 and PGC-1α to promote WAT “browning” [29]. Concurrently, rutin was observed to promote mitochondrial biogenesis in adipocytes. Given the dynamic equilibrium of mitochondria, it remains unknown whether rutin participates in regulating mitochondrial autophagy during the generation and maintenance of beige adipocytes. Given the current limited research on maintaining beige adipocyte characteristics, it is essential to investigate whether rutin serves as a mitophagy-targeting beige adipocyte maintenance agent in counteracting obesity. We evaluated rutin’s effects on beige adipocytes under cold exposure or β3-adrenergic receptor agonist stimulation, and investigated whether rutin targets mitochondrial autophagy to sustain beige induction after stimulus withdrawal.

## 2. Materials and Methods

### 2.1. Materials

MEM (M, PYG0029) and DAB Chromogenic Reagent Kit (AR1027) were purchased from BOSTER (Pleasanton, CA, USA). M5 Prestained Protein Ladder (10–180 kDa) (Mei5bio, Beijing, China; MF212-01). PrimeScript^™^ RT Master Mix (Perfect Real Time) (RR036A) and TB Green^®^ Premix Ex Taq^™^ II (Tli RNaseH Plus) (RR820A) were purchased from Takara (Kyoto, Japan). Mito-Tracker Red CMXRos (C1049B), a Mitochondrial membrane potential assay kit with JC-1 (JC-1, C2006), an ROS Assay Kit (ROS, S0033S), and an ATP Assay Kit (S0026) were purchased from Beyotime (Sganghai, China). Insulin (HY-P1156), Dexamethasone (DEX, HY-14648), Isobutylmethylxanthine (IBMX, HY-12318), CL-316243(HY-116771A), and Rutin (HY-NO148) were purchased from MCE (Monmouth Junction, NJ, USA). A Total cholesterol assay kit (TC, A111-1-1), Triglyceride assay kit (TG, A110-1-1), Alanine aminotransferase Assay Kit (ALT, C009-2-1), Aspartate aminotransferase Assay Kit (AST, C010-2-1), High-density lipoprotein cholesterol assay kit (HDL, A112-1-1), and Low-density lipoprotein cholesterol assay kit (LDL, A113-1-1) were purchased from Nanjing Jiancheng Bioengineering Institute (Nanjing, China). The following antibodies were obtained from the named sources: PRDM16 (Affinit Biosciences, Hefei, China; DF13303, 1:1000), PGC-1α (Abways, Shanghai, China; CY6630, 1:1000), UCP1 (Abclonal, Woburn, MA, USA; A5857, 1:1000), β-actin (Abclonal, Woburn, MA, USA; AC038, 1:2000), PINK1 (Abclonal, Woburn, MA, USA; A7131, 1:1000), and Parkin (BOSTER, Pleasanton, CA, USA; BM4909, 1:1000). Parkin Mouse (A76826, 1:1000), SQSTM1/p62(A95292, 1:1000), LC3B (A27590, 1:1000), and TOMM20(A78151, 1:1000) were purchased from Nature Biosciences (Nanjing, China).

### 2.2. Experimental Design and Animal Grouping

The overall experimental design is illustrated in Section 3.5. Briefly, the study consisted of two main phases: the establishment of a high-fat diet (HFD) model followed by a cold exposure and withdrawal intervention.

#### 2.2.1. HFD Obesity Model

Male C57BL/6 mice, four weeks of age, were obtained from the Shanxi Provincial People’s Hospital. The mice were housed in an environment with unrestricted access to food and water. The environment featured a 12 h light–dark cycle, 50% humidity, and a temperature range of 22 to 24 °C. Aside from temperature fluctuations, all conditions remained constant. The mice were randomly assigned to either a regular diet (*n* = 12) or a high-fat diet (HFD) (60% kcal fat, 20% carbohydrates; Medicience Ltd., Yangzhou, China) (*n* = 24) for four weeks. Subsequent HFD mice were randomly assigned to either continue on the HFD (*n* = 12) or receive the HFD supplemented with 1% (wt:wt) rutin (HFD + Rutin, *n* = 12) for eight weeks. At the same time, the ND mice maintained their original diet.

#### 2.2.2. Cold Exposure and Withdrawal Protocol

All mice (ND, HFD, and HFD + Rutin groups) were evenly randomly assigned within each group into subgroups for withdrawal cold exposure (*n* = 6) and cold exposure (*n* = 6). All mice were relocated to an artificial climate room (Shanghai Yuejin Medical Equipment Co., Ltd. Shanghai, China; HQH-Y-180), where they were subjected to a cold exposure experiment at 4 °C for 7 days. Mice in the withdrawal cold exposure group were returned to an environment with a temperature of 22–24 °C for 10 days, while those in the cold exposure group were euthanized. Each group adhered to their respective diets during the cold exposure and withdrawal cold exposure phases.

#### 2.2.3. Sample Collection and Biochemical Analysis

Mice were fasted but allowed free access to water 12 h prior to ending the cold exposure and the cold exposure withdrawal. They were anesthetized via intraperitoneal injection of sodium pentobarbital, and whole blood was collected from the orbital sinus. Serum was obtained by centrifugation for measurement of serum indicators, including TC, TG, ALT, AST, HDL, and LDL. Following blood collection, the mice were euthanized by cervical dislocation. Four tissue samples were dissected: epididymal WAT (eWAT) from the epididymis and surrounding testicular area, iWAT from the subcutaneous groin region, BAT from the interscapular fat depot, and liver tissue. One portion was fixed in 4% paraformaldehyde and then embedded in paraffin, while the remaining samples were stored at low temperatures. All experiments were approved by the **Scientific Research** Ethics Committee of Fenyang College, Shanxi Medical University (Number: 2025003).

### 2.3. Cell Culture and Treatment

3T3L-1 cells, also known as mouse embryonic fibroblasts, were purchased from the China Center for Type Culture Collection (Wuhan, China). Undifferentiated preadipocyte 3T3-L1 cells were cultured in complete medium (MEM, 10% FBS and 1% tris-buffered saline). Cells were cultured at 37 °C with 5% CO_2_ until they reached 70–80% confluence. After Induction Medium I (complete medium supplemented with 0.5 mM IBMX, 1 μM DEX, and 10 μg/mL insulin) was added to the cells for 3 days, a switch to Induction Medium II (complete medium supplemented with 10 μg/mL insulin) was carried out for 2 days. Following this, complete medium was added to the cells for 2 days to maintain lipid droplet formation. To establish a beige adipocyte model, mature adipocytes were cultured for two days in complete medium supplemented with 1 μM CL. Subsequently, various parameters of the beige adipocytes were assessed after removing 1 μM CL for 3 days. Based on our preliminary evidence indicating that 60 μM rutin upregulates UCP1 and PGC-1α without cytotoxicity [29], this concentration was selected for further experiments. A simultaneous addition of 1 μM CL and 60 μM rutin was made to the complete medium for a duration of 2 days. Following the withdrawal of CL, beige adipocytes continued to receive treatment with 60 μM rutin.

### 2.4. IPGTT

Following 16 h of food deprivation with unrestricted access to water, glucose tolerance tests were performed on both groups of mice at 5:00 PM on the day before the fifth day of cold exposure and again on the seventh day after withdrawal from cold exposure. Blood samples were obtained from the tail vein to assess fasting blood glucose levels, the time designated as 0 min. Subsequently, the mice received an intraperitoneal injection of 20% glucose at a dosage of 2 g/kg. Blood glucose levels were recorded at 30, 60, 90, and 120 min post-injection.

### 2.5. H&E and IHC Staining

After baking at 60 °C for 1 h, paraffin sections underwent deparaffinization in xylene and were hydrated through a graded ethanol series. The sections were then stained with hematoxylin for nuclear staining and differentiated with 1% hydrochloric acid alcohol. Then eosin solution was used for H&E staining.

After routine dewaxing to water, experiments were performed according to the immunohistochemistry kit instructions. The following primary antibody dilutions were used: UCP1 (1:200), TOMM20 (1:100), and PINK1 (1:100), and the experiments were performed overnight at 4 ° C, followed washing with PBS for 5 min. The secondary antibody was added, incubated at 37 ° C for 30 min, and then washed with PBS. SABC was then added, incubated at 37 ° C for 30 min, and washed with PBS. DAB developing solution was pipetted onto sections under the microscope for color development. Acid–alcohol differentiation was performed. Rehydration and clearing treatments were conducted. Finally, sections were examined under a microscope.

### 2.6. Western Blot Analysis

Samples were lysed in ice-cold RIPA buffer supplemented with 1% (*v*/*v*) PMSF to obtain proteins. Proteins were denatured by boiling them in 5× SDS sample buffer. Based on the varying molecular weights of the proteins, SDS–polyacrylamide gels with concentrations of 10% or 12.5% were utilized for electrophoretic separation. Following separation, the gels were transferred to nitrocellulose membranes. The membranes were blocked at room temperature with 5% skimmed milk powder, after which specific protein antibodies were incubated at 4 °C overnight. Subsequently, the horseradish peroxidase (HRP)-conjugated goat anti-rabbit IgG antibody (BOSTER, Pleasanton, CA, USA; BA1054, 1:2000) was applied to the membranes at room temperature for 2 h. Finally, protein images were visualized using a chemiluminescence.

### 2.7. qPCR

Total RNA was extracted from samples using TRIzol. A commercial kit was used to reverse-transcribe the resulting cDNA, and qPCR was then conducted. The Section 2 contains information on the kit. Lastly, gene expression levels were measured using the 2^−^^ΔΔCT^ technique, with β-actin serving as a control. All primers were designed and synthesized by Sangon Biotech (Shanghai) Co., Ltd. (Shanghai, China). All gene sequences are shown in Table 1.

**Table 1:** Primer sequences used in this study.

Target Gene	Forward Primer (5′-3′)	Reverse Primer (5′-3′)
PINK1	GAGGAGAAGCAGGCGGAAGG	TGCCAGCATCGAGTGTCCAG
Parkin	TTGACACGAGTGGACCTGAGC	ACCTCTGGCTGCTTCTGAATCC
β-actin	GTGCTATGTTCTAGACTTCG	ATGCCACAGGATTCCATACC

### 2.8. Oil Red O Staining

For culture, seed 3T3-L1 cells into a 6-well plate. Follow the staining directions provided by the Oil Red O kit’s manufacturer (Solaibio, Beijing, China; G1262). Lastly, cover the cells with a tiny bit of distilled water and use a microscope to take pictures.

### 2.9. Immunofluorescence

Cells were seeded into a 24-well plate for intervention using the same method and then fixed with 4% paraformaldehyde. Subsequently, 0.2% Triton X-100 was added for 1 h. After washing with PBS, 5% goat serum was added. Diluted primary antibody working solutions (UCP1 at 1:200, Parkin at 1:100, Tomm20 at 1:100) were directly applied to the samples. Then they were incubated overnight at 4 °C. For the Parkin/TOMM20 co-staining, each primary antibody was incubated separately overnight at 4 °C. Following this, a species-specific fluorescent secondary antibody (1:50 dilution) was added for 1 h. Finally, DAPI nuclear stain (BOSTER, Pleasanton, CA, USA; AR1176) was added for 30 min, and images were acquired using a microscope.

### 2.10. Mito-Tracker Red Staining

The Mito-Tracker Red CMXRos stock solution (200 μM) was diluted with complete medium in the proper ratio to create a working solution with a final concentration of 200 nM. The cells were incubated in the working solution for 30 min. After fixation with 4% paraformaldehyde, 0.2% Tween-20 was added for one hour. Cell nuclei were stained with DAPI for half an hour. Lastly, the samples were viewed under a fluorescent microscope after being covered with the complete medium.

### 2.11. ATP Detection

Add the lysis buffer from the ATP assay kit to the cells in a 6-well plate and lyse them on ice. Centrifuge and collect the supernatant for later use. A standard curve was established by diluting the ATP standard in lysis buffer to create a series of concentrations, according to the manufacturer’s protocol. Prepare the ATP assay working solution by diluting ATP assay reagent and ATP assay diluent at a 1:9 ratio, based on the requirement of 100 μL working solution per sample. Dispense the ATP working solution into an opaque 96-well black plate. After 3–5 min, add 20 μL of standard or sample to each well. Determine RLU values using chemiluminescence in an ELISA reader while simultaneously measuring protein concentration via BCA assay. Convert units to nmol/mg by dividing ATP concentration by BCA concentration. Perform triplicate replicates per group, calculate the mean, divide by the mean of the control group, and determine the relative ATP yield.

### 2.12. ROS

After the treatment, the cells were digested and collected in centrifuge tubes. Then a 10 μmol/L DCFH-DA solution was prepared by diluting DCFH-DA in serum-free medium at a ratio of 1:1000 and added to the cells. During the incubation, the tubes were inverted every 5 min to promote thorough mixing. The cells were washed with serum-free medium before performing flow cytometric analysis.

### 2.13. JC-1

JC-1 (200× stock solution) was added to ultrapure water and shaken vigorously to ensure thorough mixing. The JC-1 buffer (5×) was incorporated. This completed the preparation of the JC-1 staining working solution. The JC-1 staining buffer (5×) was then diluted with distilled water to obtain the JC-1 staining buffer (1×). After treatment, the culture medium was removed from the well plate. The JC-1 working solution was added to the well plate and incubated at 37 °C for 20 min. The cells were washed with the JC-1 staining buffer (1×). Finally, the cells were covered with medium and observed.

### 2.14. Statistical Analysis

Statistical analysis of experimental results was performed using ImageJ and GraphPad Prism 10.0 software. Data are presented as means ± SEMs. For all data that met the assumptions for parametric testing, normality was assessed using the Shapiro–Wilk test and homogeneity of variance was evaluated using Levene’s test. Inter-group comparisons were analyzed using one-way ANOVA or two-way ANOVA. Tukey’s test was used for post hoc multiple comparisons. *p* < 0.05 was considered statistically significant.

## 3. Results

### 3.1. Rutin Inhibited the Beige-to-White Transition and Preserved the Phenotype of Beige Adipocytes Following CL Withdrawal

**Figure 1:**
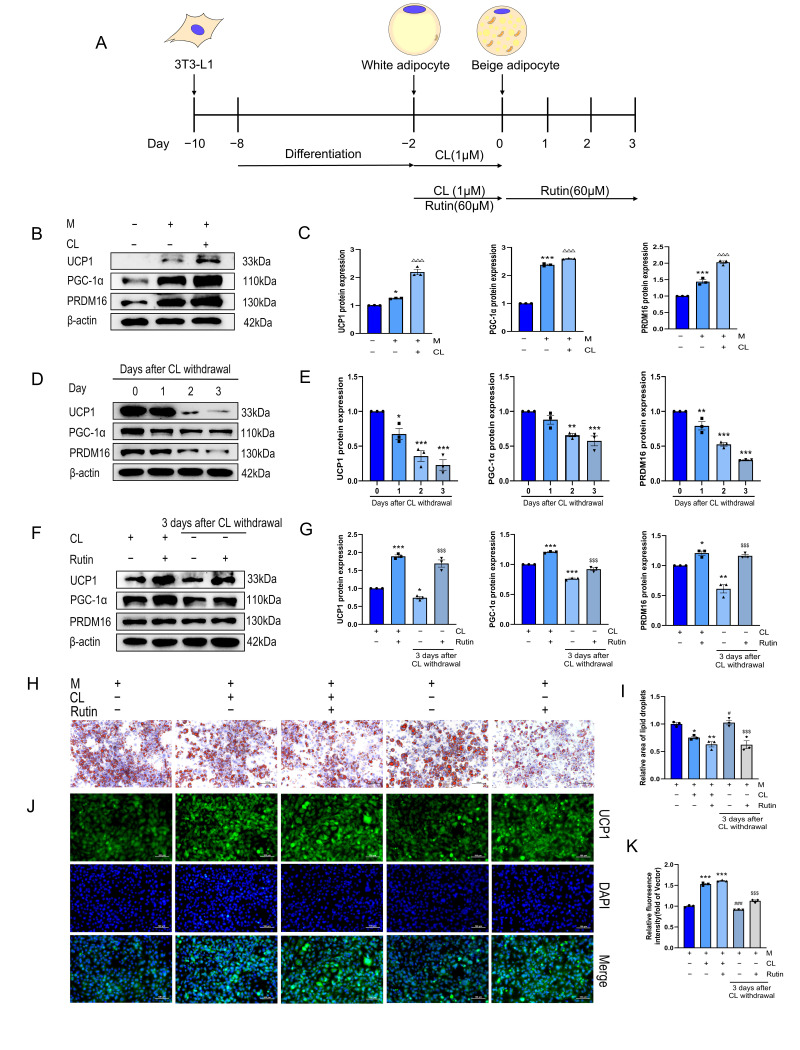
Rutin maintains beige adipocyte characteristics. *n* = 3. (**A**) Schematic diagram of in vitro experiments. (**B**,**C**) Expression and analysis of thermogenic proteins in beige induction models. (**D**,**E**) Expression and analysis of thermogenic proteins in beige adipocytes 3 days after CL withdrawal. (**F**,**G**) Effects of rutin on thermogenic proteins in beige adipocytes and analysis. (**H**,**I**) Oil Red O staining and data analysis. Scale bar is 100 µm. (**J**,**K**) UCP1 immunofluorescence staining and data analysis. Scale bar is 100 µm. Data represent the means ± SEMs. Significance was determined by one-way ANOVA followed by Tukey’s post hoc test for multiple comparisons. Statistical significance is represented as follows: * *p* < 0.05, ** *p* < 0.01, *** *p* < 0.001 vs. control group; ^△△△^ *p* < 0.001 vs. white adipocyte group; ^#^ *p* < 0.05, ^###^ *p* < 0.001 vs. +CL group; ^$$$^ *p* < 0.001 vs. −CL group.

A beige induction model was established by treating differentiated 3T3-L1 adipocytes with 1 μM CL for two days. This elevated the protein expression of the beige-specific markers UCP1, PGC-1α, and PRDM16 (Figure 1B,C). The expression of these proteins progressively declined over three days (Figure 1D,E) following CL withdrawal, consistent with the reported instability of the beige phenotype [20].

Co-treatment with CL and rutin for two days also upregulated the expression of thermogenic protein (Figure 1F,G). Rutin sustained the expression of these thermogenic proteins after CL withdrawal. Oil Red O staining results further indicated that the CL–rutin combination improved adipocyte morphology and attenuated lipid droplet accumulation. This effect persisted with rutin alone after withdrawal (Figure 1H,I). Immunofluorescence analysis confirmed that rutin synergized with CL to enhance UCP1 expression. Following CL withdrawal, UCP1 fluorescence intensity remained higher in rutin-maintained adipocytes (-CL + R) than in those without rutin (-CL) (Figure 1J,K). These results demonstrate that rutin mitigates the reversion of beige adipocytes to a white phenotype and helps maintain their characteristic features in vitro.

### 3.2. Rutin Alleviated Mitochondrial Dysfunction in Beige Adipocytes Following CL Withdrawal

**Figure 2:**
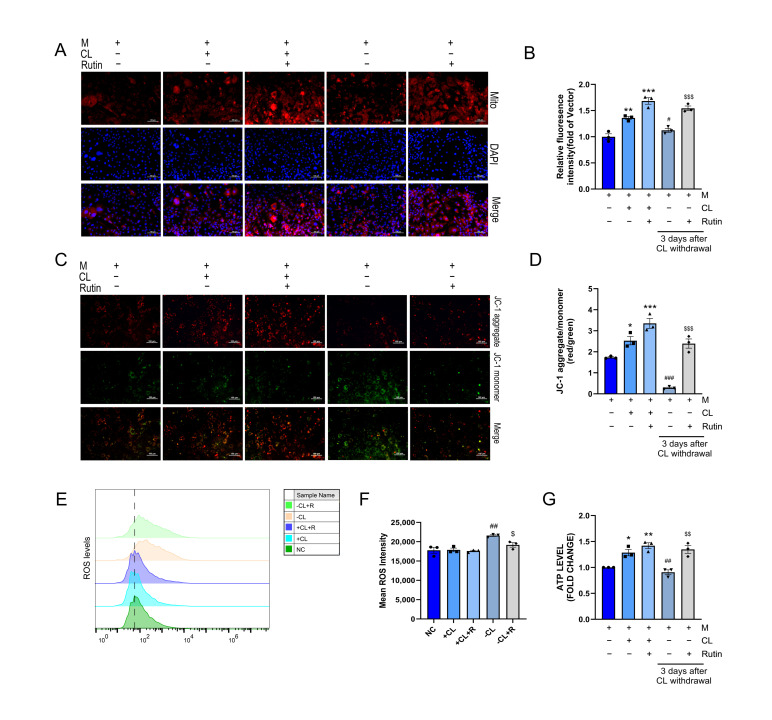
Rutin maintains mitochondrial function. *n* = 3. (**A**,**B**) Mito-Tracker staining and fluorescence intensity analysis. (**C**,**D**) JC-1 staining and fluorescence intensity analysis. (**E**,**F**) ROS flow cytometry analysis. (**G**) ATP detection analysis. Scale bar is 100 µm. Data represent the means ± SEMs. Significance was determined by one-way ANOVA followed by Tukey’s post hoc test for multiple comparisons. Statistical significance is represented as follows: * *p* < 0.05, ** *p* < 0.01, *** *p* < 0.001 vs. control group; ^#^ *p* < 0.05, ^##^ *p* < 0.01, ^###^ *p* < 0.001 vs. +CL group; ^$^ *p* < 0.05, ^$$^ *p* < 0.01, ^$$$^ *p* < 0.001 vs. −CL group.

Mitochondrial functional stability is necessary for beige adipocyte maintenance. Mito Tracker staining showed that rutin induced mitochondrial biogenesis and reduced mitochondrial loss persistently after CL withdrawal (Figure 2A,B). Continuous administration of rutin increased the mitochondrial membrane potential (MMP) that protected against mitophagy induced by depolarization, which was evidenced by increased JC-1 red fluorescent aggregates (Figure 2C,D). While reactive oxygen species (ROS) levels were unaffected by CL and rutin co-treatment, a significant increase was observed after CL withdrawal; this increase was suppressed by continued rutin intervention (Figure 2E,F). Despite the high UCP1 expression typically associated with mitochondrial uncoupling, both CL and rutin increased cellular ATP levels (Figure 2G). In conclusion, rutin maintains mitochondrial functional stability, a key mechanism in preventing the transition from beige to white adipocytes, thereby identifying a potential target for combating obesity.

### 3.3. Rutin Suppressed Mitochondrial Autophagy in Beige Adipocytes After CL Withdrawal

**Figure 3:**
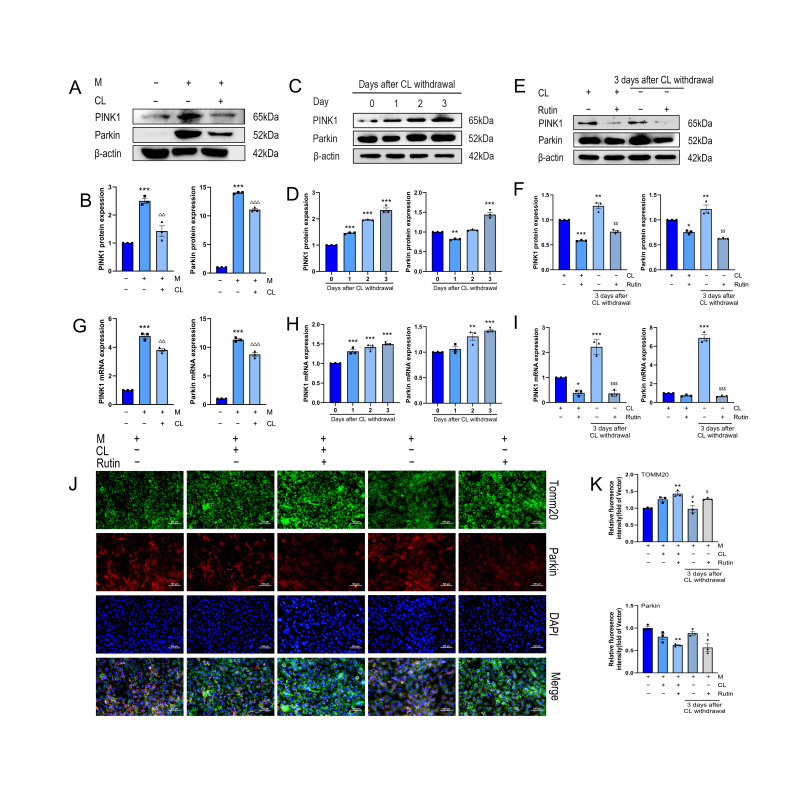
Rutin inhibits mitochondrial autophagy in beige adipocytes. *n* = 3. (**A**,**B**) Expression and analysis of mitophagy proteins in beige induction model. (**C**,**D**) Expression and analysis of mitophagy proteins in beige-to-white transition model. (**E**,**F**) Effects and analysis of rutin on mitophagy proteins in beige adipocytes. (**G**–**I**) RT-qPCR detection of mitophagy genes PINK1 and Parkin mRNA. (**J**,**K**) Immunofluorescence staining of mitochondrial marker TOMM20 and Parkin. Scale bar is 100 µm. Data represent the means ± SEMs. Significance was determined by one-way ANOVA followed by Tukey’s post hoc test for multiple comparisons. Statistical significance is represented as follows: * *p* < 0.05, ** *p* < 0.01, *** *p* < 0.001 vs. control group; ^△△^ *p* < 0.01, ^△△△^ *p* < 0.001 vs. white adipocytes group; ^#^ *p* < 0.05 vs. −CL group; ^$^ *p* < 0.05, ^$$^ *p* < 0.01, ^$$$^ *p* < 0.001 vs. −CL group.

We established models of both beige induction (Figure 3A,B) and beige-to-white transition (Figure 3C,D). Western blot analysis of the canonical mitophagy markers PINK1 and Parkin confirmed that beige-to-white transition is associated with enhanced mitophagy.

We next examined whether rutin influences mitophagy during this phenotypic transition. Western blot (Figure 3E,F) and PCR (Figure 3G–I) analyses demonstrated that rutin downregulated the expression of PINK1 and Parkin. Consistent with this, immunofluorescence co-staining for the mitochondrial marker TOMM20 and Parkin showed that rutin increased TOMM20 intensity while reducing Parkin signaling (Figure 3J,K), further supporting its suppressive effect on mitophagy.

### 3.4. The Changes in LC3B/p62 Protein Expression of Beige Adipocytes After Rutin Treatments Were Consistent with Autophagy Activity

**Figure 4:**
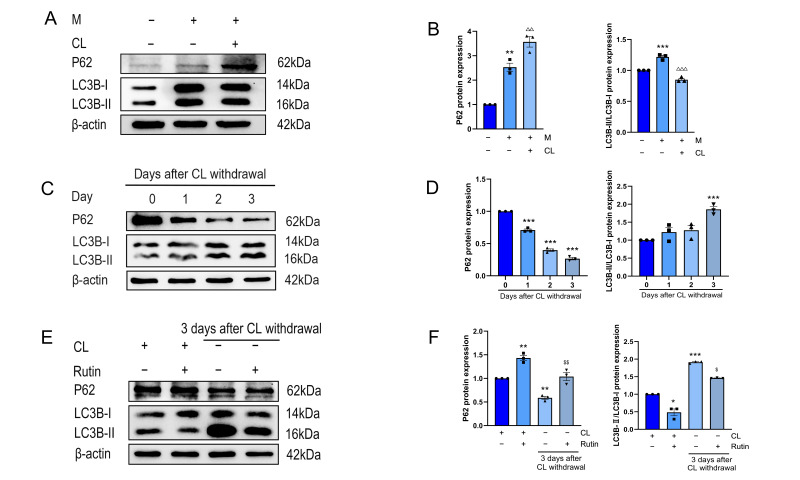
Rutin affects the level of LC3B/P62in beige adipocytes. *n*=3. (**A**,**B**) Western blot expression and analysis of P62 and LC3B in beige induction models. (**C**,**D**) Expression and analysis of P62 and LC3B proteins in beige-to-white transition model. (**E**,**F**) Effects of rutin on P62 and LC3B protein expression in beige adipocytes and analysis. Data represent the means ± SEMs. Significance was determined by one-way ANOVA followed by Tukey’s post hoc test for multiple comparisons. Statistical significance is represented as follows: * *p* < 0.05, ** *p* < 0.01, *** *p* < 0.001 vs. control group; ^△△^ *p* < 0.01, ^△△△^ *p* < 0.001 vs. white adipocytes group; ^$^ *p* < 0.05, ^$$^ *p* < 0.01 vs. −CL group.

We observed decreased microtubule-associated proteins 1A/1B light chain 3B (LC3B)-II during beige adipocyte differentiation (Figure 4A,B), whereas CL withdrawal increased LC3B-II and decreased P62, consistent with activated mitophagy (Figure 4C,D). Rutin treatment reversed this pattern, inhibiting LC3B-II expression and promoting P62 accumulation (Figure 4E,F). The reduction in LC3B-II after rutin treatment indicated inhibition of autophagosome formation. Increased P62 expression suggests that Parkin-dependent upstream parkin ubiquitination is impaired. In conclusion, the expression of LC3B/p62 was accordant with the inhibition of mitochondrial autophagy after rutin intervention in the beige-to-white transition model.

### 3.5. Rutin Continuously Improved Metabolic Indicators in HFD-Induced Obese Mice After Withdrawal of Cold Stimulation

**Figure 5:**
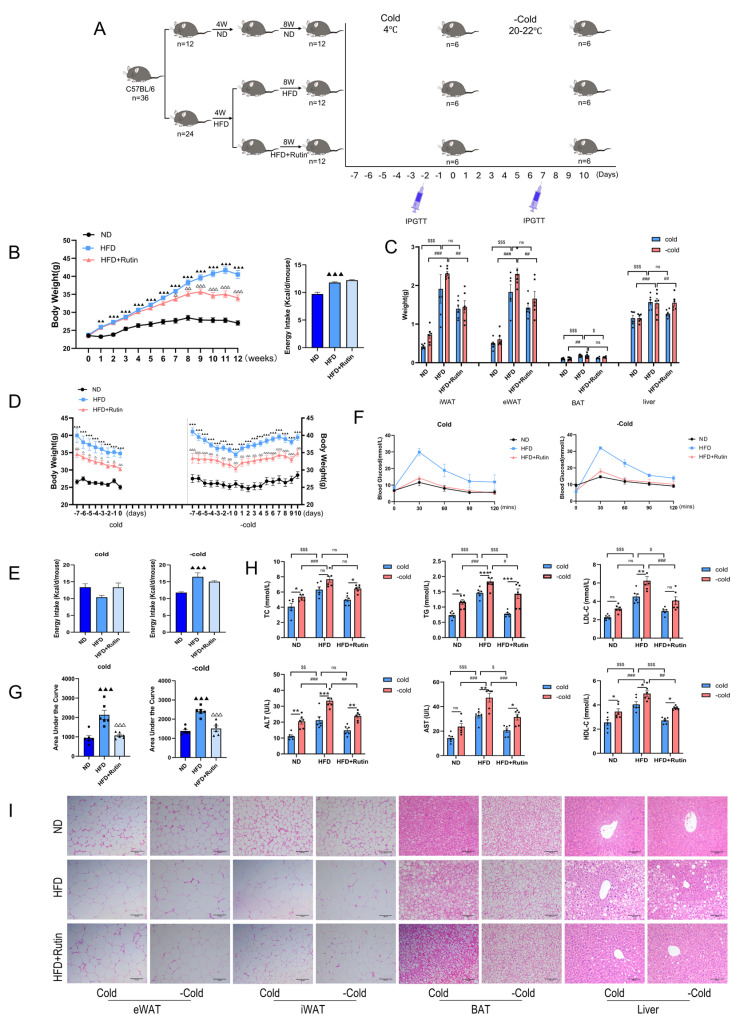
Rutin continued to improve metabolic parameters in mice with high-fat-diet-induced obesity after cold exposure was stopped. *n* = 6. (**A**) Schematic of in vivo experiments. High-fat-diet-induced obese mouse model, cold exposure withdrawal mouse model, and IPGTT injection timing. (**B**) Body weight and food intake of high-fat-diet-induced obese mice. (**C**) Tissue weights of eWAT, iWAT, BAT, and liver. (**D**,**E**) Body weight and food intake of cold exposure withdrawal model mice. (**F**,**G**) Intraperitoneal glucose tolerance curve and area under the curve in the cold exposure withdrawal model. (**H**) Plasma TC, TG, HDL, LDL, ALT, and AST levels. Dots represent individual mice per group. (**I**) H&E staining of eWAT, iWAT, BAT, and liver tissues. Scale bar is 100 µm. Dots represent individual mice per group. Data represent the means ± SEMs. The comparison of Cold and -Cold conditions was analyzed by two-way ANOVA and that of other conditions by one-way ANOVA, followed by Tukey’s post hoc test for multiple comparisons. Statistical significance is represented as follows: ns indicates no significant difference; ^▲▲^ *p* < 0.01, ^▲▲▲^ *p* < 0.001 vs. ND group; ^△^ *p* < 0.05, ^△△^ *p* < 0.01, ^△△△^ *p* < 0.001 vs. HFD group; * *p* < 0.05, ** *p* < 0.01, *** *p* < 0.001 vs. Cold group; ^#^ *p* < 0.05, ^##^ *p* < 0.01, ^###^ *p* < 0.001 vs. HFD + −Cold group; ^$^ *p* < 0.05, ^$$^ *p* < 0.01, ^$$$^ *p* < 0.001 vs. HFD + Cold group.

We first established a diet-induced obese mouse model. Compared to ND mice, HFD mice showed a significantly higher body weight from the first week onward, despite greater food intake (Figure 5B). Rutin supplementation (HFD + Rutin) persistently suppressed body weight gain starting from the third week of treatment, without affecting food consumption.

During a 7-day cold exposure period, all groups lost weight. HFD mice maintained higher body weights than ND mice, whereas HFD + Rutin mice remained lighter than HFD controls (Figure 5D). Food intake did not differ significantly among groups during cold exposure. Ten days after returning to thermoneutrality, HFD mice consumed more calories than ND mice, though not significantly more than the HFD + Rutin group (Figure 5E). This suggests that rutin helps maintain metabolic balance post-cold. Rutin also reduced the weights of iWAT and eWAT after cold withdrawal, but not those of BAT or liver tissues (Figure 5C).

Intraperitoneal glucose tolerance tests showed that HFD mice developed glucose intolerance, which was significantly ameliorated by rutin treatment (Figure 5F,G). Serum analyses further revealed that rutin attenuated cold-withdrawal-induced dyslipidemia (TC, TG, HDL, and LDL) and improved liver function markers (ALT and AST) in HFD mice (Figure 5H). H&E staining revealed that rutin decreased adipocyte size and hepatic lipid vacuolation after cold withdrawal, which is consistent with these metabolic enhancements (Figure 5I).

### 3.6. Rutin Preserved the Thermogenic Characteristics of Beige Adipocytes in the iWAT Following Withdrawal of Cold Stimulation

**Figure 6:**
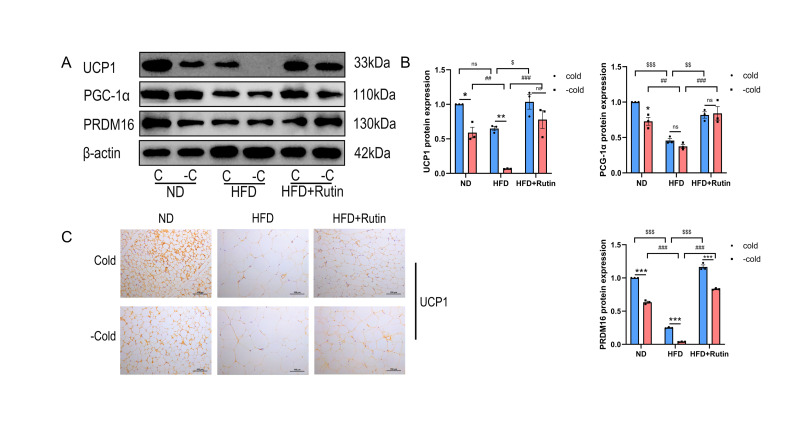
Rutin preserves the molecular characteristics of beige adipocytes following withdrawal from cold exposure. (**A**,**B**) Western blot analysis and interpretation of brown adipocyte proteins UCP1, PGC-1α and PRDM16 in ND, HFD, and HFD + Rutin groups under cold exposure and withdrawal conditions. n = 3. (**C**) UCP1 immunohistochemistry in ND, HFD, and HFD + Rutin groups during cold exposure and withdrawal. Scale bar is 100 μm. Data represent the means ± SEMs. Significance was determined by two-way ANOVA followed by Tukey’s post hoc test for multiple comparisons. Statistical significance is represented as follows: ns indicates no significant difference; * *p* < 0.05, ** *p* < 0.01 *** *p* < 0.001 vs. Cold group; ^##^ *p* < 0.01, ^###^ *p* < 0.001 vs. HFD + −Cold group; ^$^ *p* < 0.05, ^$$^ *p* < 0.01, ^$$$^ *p* < 0.001 vs. HFD + Cold group.

To assess whether rutin influences beige adipocyte maintenance in vivo, we examined the protein expression of key thermogenic markers. Rutin administration sustained the expression of these proteins both during cold exposure and after its withdrawal, indicating a stabilizing effect on the beige phenotype following the cessation of cold stimulus (Figure 6A,B). Immunohistochemical analysis of UCP1 further supported these findings: during cold exposure, UCP1 positivity was highest in the ND group, intermediate in the HFD + Rutin group, and lowest in the HFD group. The HFD + Rutin group maintained significantly higher UCP1 levels than the HFD group (Figure 6C) after cold withdrawal. In summary, these results demonstrate that rutin not only promotes beige adipocyte formation in HFD-fed mice iWAT but also mitigates their loss upon withdrawal of cold stimulation.

### 3.7. Rutin Suppressed Mitochondrial Autophagy Following Cold Withdrawal and Increased Mitochondrial Numbers in iWAT

**Figure 7:**
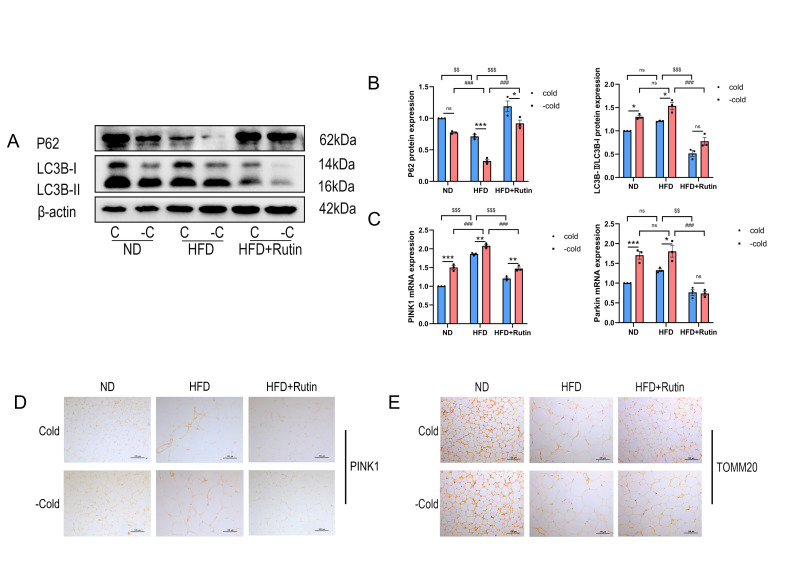
Rutin continuously suppressed mitochondrial autophagy in iWAT following cold withdrawal while increasing mitochondrial number in iWAT. *n* = 3. (**A**,**B**) Analysis of P62 and LC3B protein expression and autophagy flux in iWAT mitochondria from cold exposure withdrawal model mice. (**C**) qPCR detection of PINK1 and Parkin mRNA, autophagy-related genes in iWAT mitochondria. (**D**) Immunohistochemistry for the mitochondrial autophagy protein PINK1 in iWAT. (**E**) Immunohistochemistry for the mitochondrial marker protein TOMM20 in iWAT. Scale bar is 100 μm. Data represent the means ± SEMs. Significance was determined by two-way ANOVA followed by Tukey’s post hoc test for multiple comparisons. Statistical significance is represented as follows: ns indicates no significant difference; * *p* < 0.05, ** *p* < 0.01 *** *p* < 0.001 vs. Cold group; ^###^ *p* < 0.001 vs. HFD + −Cold group; ^$$^ *p* < 0.01, ^$$$^ *p* < 0.001 vs. HFD + Cold group.

According to Western blot examination, rutin reduced LC3B-II levels and raised P62, suggesting a compromised autophagic flow (Figure 7A,B). Reduced PINK1 and Parkin mRNA expression, as well as decreased PINK1 protein detection by immunohistochemistry, further corroborated this suppression (Figure 7C,D). We measured TOMM20 expression to see if rutin’s impact on mitophagy affected mitochondrial content. Rutin improved TOMM20 staining in iWAT after cold exposure and removal, according to immunohistochemical analysis (Figure 7E), indicating intact mitochondrial mass. Rutin treatment significantly changed LC3B/p62 levels consistently with reduced autophagy activity, which is consistent with our in vitro research results.

## 4. Discussion

Our research first addresses the question of whether rutin can sustain the beige adipocyte state. In this study, we demonstrated that rutin promotes the upregulation of UCP1 and the expression of beige adipogenesis-promoting factors PRDM16 and PGC-1α in both 3T3-L1 cells and in iWAT under cold and CL treatment. Furthermore, it maintains the characteristics of beige adipocytes after cold exposure or CL withdrawal (Figure 1).

Continued rutin addition after the removal of external stimuli failed to return beige adipocyte-related gene expression to pre-withdrawal levels. However, compared to untreated cells after stimulus withdrawal, the expression levels exhibited significant differences. Thus, our hypothesis was confirmed: rutin can sustain the retention of beige adipocyte characteristics.

Cold exposure promotes beige adipocyte formation primarily through sympathetic norepinephrine release and subsequent β3-adrenergic receptor (β3-AR) activation, which initiates an AMPK-PKA-CREB signaling cascade to upregulate thermogenic gene expression [30]. The β3-AR agonist CL is commonly used to mimic this cold-induced browning process. As beige adipocyte activation requires mitochondrial biogenesis and functional remodeling, we first evaluated MMP using JC-1 staining. Our results demonstrate that rutin enhanced MMP in CL-stimulated beige adipocytes and sustained its stability after CL withdrawal. This suggests that rutin has a protective effect on MMP during and after β3-adrenergic stimulation.

Cold exposure helps maintain mitochondrial function by modulating ROS at physiologically appropriate levels. In our experiments, CL treatment alone did not significantly alter ROS levels in 3T3-L1-derived beige adipocytes, whereas rutin supplementation reduced ROS, an effect that persisted after CL withdrawal. This finding suggests that while CL intervention promotes beige adipocyte formation and improves mitochondrial function, it may cause some damage to mitochondrial function upon CL withdrawal. Rutin intervention not only preserves beige adipocyte existence but also maintains the stability of mitochondrial function-related indicators. 

UCP1 uncouples cellular respiration from mitochondrial ATP synthesis and leads to energy dissipation as heat [31]. Cold-induced activation of β3-AR and α1-AR elevates intracellular Ca^2+^ via SERCA2b and RyR2 [32]. These Ca^2+^ are subsequently taken up into mitochondria, where they activate PDP1c, enhance pyruvate dehydrogenase activity, and stimulate ATP synthesis [32]. Notably, consistent with our previous findings that rutin promotes beige induction via the CaMKKβ-AMPK pathway [29], the current study showed that rutin increased and sustained ATP levels in beige adipocytes while also upregulating UCP1. Based on these results, we hypothesize that rutin may exert its browning effects partially through the ATP-dependent calcium cycling pathway, while the upregulation of UCP1 may involve other signaling mechanisms. It may also be related to compensatory mechanisms triggered by cold induction simulated by CL. 

This study showed that rutin treatment effectively reduced body weight in HFD mice. This finding is consistent with previous reports [29]. Rutin also improved metabolic disorders and reduced adipose tissue hypertrophy in HFD mice after cold exposure withdrawal. Then we examined the maintenance effect of rutin on beige adipocytes in iWAT. We measured the expression of the beige adipocyte marker proteins UCP1, PRDM16, and PGC-1α in iWAT and performed UCP1 immunohistochemistry. Consistent with the results in 3T3-L1 cells, rutin maintained the thermogenic phenotype of beige adipocytes in iWAT after cold exposure withdrawal. In conclusion, these studies preliminarily demonstrate that rutin acts as a beige maintenance agent against HFD-induced obesity in mice. Although we were unable to assess the energy metabolism level of each mouse directly, this conclusion is supported by measurements of energy intake and related metabolic parameters, such as TC and TGs.

Next, we explored the mechanism by which rutin maintains beige adipocyte characteristics. Previous studies have shown that during the transition from brown to white adipocytes, PINK1-mediated mitophagy contributes to the loss of thermogenic capacity by eliminating mitochondria [33], suggesting that suppressing mitophagy may help preserve the beige phenotype. Both in 3T3-L1 cells and in iWAT, it was revealed that rutin reduced the protein and mRNA expression of PINK1 and Parkin. At the same time, changes in LC3B/P62 levels consistent with decreased autophagic activity were detected both in 3T3-L1 cells and in iWAT. We verified the expression of mitochondrial autophagy in both in 3T3-L1 cells and in iWAT. This strongly demonstrated that rutin maintains the thermogenic phenotype of beige adipocytes accompanied by the suppression of PINK1/Parkin-mediated mitochondrial autophagy. 

Building upon previous findings that rutin upregulates UCP1, this study further reveals that it not only acts synergistically with CL to enhance beige adipocyte marker expression but also sustains the beige phenotype after CL withdrawal. We demonstrated that rutin maintains beige adipocyte characteristics by preserving mitochondrial function and concomitantly suppressing mitophagy, thereby preventing beige-to-white transition following the cessation of cold or β-adrenergic stimulation. Importantly, rutin also ameliorated metabolic disturbances in diet-induced obese mice after cold withdrawal. While research on strategies to maintain beige adipocytes for obesity treatment remain limited, our findings not only elucidate a mechanism by which rutin acts as a mitophagy-targeting beige-maintenance agent, but also offer a promising therapeutic avenue for obesity management. The main findings of this study are based on 3T3-L1 cells and mouse models. Further validation is required for the application of rutin in humans, and this research strongly supports its potential therapeutic value.

## 5. Conclusions

In this study, we used cell and animal models to demonstrate that rutin maintains the thermogenic properties of beige adipocytes after the withdrawal of cold stimulation. Our findings show that rutin inhibits beige-to-white transition while preserving mitochondrial function and suppressing PINK1/Parkin-mediated mitophagy. Changes in LC3B/P62 levels during this process were consistent with mitophagy activity. Together, these results indicate that rutin acts as a mitophagy-targeting beige-maintenance agent, suggesting a new potential therapeutic direction for obesity.

## Data Availability

The original contributions presented in the study are included in the article. Further inquiries can be directed to the corresponding author.

## Abbreviations

The following abbreviations are used in this manuscript:

WAT	White adipocyte tissue
BAT	Brown adipocyte tissue
eWAT	Epididymal white adipose tissue
iWAT	Inguinal white adipose tissue
UCP1	Uncoupling protein-1
PGC-1α	Peroxisome proliferator-activated receptor γ coactivator 1alpha
PRDM16	PR domain-containing 16
LC3	Microtubule-associated protein light chain 3
LC3B-II	Microtubule-associated proteins 1A/1B light chain 3B-II
P62	Sequestosome 1
TOMM20	Translocase of outer mitochondrial membrane 20
RT-qPCR	Quantitative real-time PCR
ROS	Reactive oxygen species
ATP	Adenosine triphosphate
MMP	Mitochondrial membrane potential
JC-1	Mitochondrial membrane potential assay kit with JC-1
M	Minimum essential medium, MEM
H&E	Hematoxylin and eosin
HFD	High-fat diet
